# The Impact of Resilience and Extraversion on Psychological Distress, Loneliness, and Boredom During the COVID-19 Pandemic: A Follow-Up Study Among the General Population of Tyrol, Austria

**DOI:** 10.3389/fpsyt.2021.777527

**Published:** 2021-11-12

**Authors:** Franziska Tutzer, Beatrice Frajo-Apor, Silvia Pardeller, Barbara Plattner, Anna Chernova, Christian Haring, Bernhard Holzner, Georg Kemmler, Josef Marksteiner, Carl Miller, Martin Schmidt, Barbara Sperner-Unterweger, Alex Hofer

**Affiliations:** ^1^Department of Psychiatry, Psychotherapy and Psychosomatics, Division of Psychiatry I, Medical University Innsbruck, Innsbruck, Austria; ^2^Department of Psychiatry, General Hospital of Bolzano, Sanitary Agency of South Tyrol, Bolzano, Italy; ^3^Department of Psychiatry and Psychotherapy B, State Hospital Hall in Tyrol, Hall in Tyrol, Austria; ^4^Department of Psychiatry and Psychotherapy A, State Hospital Hall in Tyrol, Hall in Tyrol, Austria; ^5^Department of Psychiatry, County Hospital Kufstein, Kufstein, Austria; ^6^Department of Psychiatry, County Hospital Lienz, Lienz, Austria; ^7^Department of Psychiatry, Psychotherapy and Psychosomatics, Division of Psychiatry II, Medical University Innsbruck, Innsbruck, Austria

**Keywords:** COVID-19, pandemic, psychological distress, loneliness, boredom, resilience, extraversion

## Abstract

**Background:** The Covid-19 pandemic and related measures represent an enormous burden on mental health. The aim of this study was to investigate longitudinal changes in psychological distress, loneliness, boredom, and resilience over the course of the pandemic and to examine the associations between resilience and extraversion at baseline (summer 2020) and psychological distress, loneliness, and boredom at 5-month-follow-up.

**Methods:** Residents of Tyrol (≥18a) completed an online survey on psychological distress, loneliness, boredom, resilience, and extraversion by using the Brief-Symptom-Checklist, the Three-Item Loneliness Scale, the Multidimensional State Boredom Scale-Short Form (MSBS-SF), the Resilience Scale, and the Extraversion subscale of the Big Five Inventory.

**Results:** Of the 961 baseline participants, 384 took part in the follow-up survey. The percentage of study participants with striking psychological distress remained the same. Similarly, resilience did not change from baseline to follow-up, whereas the number of those experiencing moderate loneliness increased significantly. In contrast, at follow-up, severe loneliness was detected in significantly less people. Boredom decreased significantly over time. A moderate negative association was detected between baseline resilience and psychological distress, loneliness, and boredom at follow-up, and a weak but still significant negative association between extraversion and these outcomes.

**Discussion:** These findings indicate that a subset of the general population consistently suffers from high levels of psychological distress and point to the protective effects of resilience and extraversion in this context. They reemphasize the importance of prevention and mitigation strategies to address these public health problems.

## Introduction

The COVID-19 pandemic and related measures represent an enormous burden on mental health (1) and have been associated with increased levels of anxiety, depression, frustration, insecurity, agitation, sleep disturbances, and boredom (2). Boredom itself, in turn, promotes depressive and anxiety symptoms, and bored people are at a higher risk of using drugs (3, 4). Similarly, public health policy measures may aggravate loneliness, which is a further risk factor for mental and physical health problems (5) and has been associated with suicidal thoughts (6).

Still, people respond differently to challenges and difficulties like the COVID-19 pandemic and there are not only risk factors but also protective factors in regards of mental health, e.g., resilience and extraversion. Resilience refers to the ability to recover from disadvantages, adapt positively, and defy adversity (7) and has been associated with psychological well-being (8). Extraversion is a personality trait that describes people who are assertive and talkative, seek social closeness, and enjoy making new contacts (9, 10). They experience new and complex events at a lower stress level and with more positive feelings and are therefore more resistant to stressful situations (11, 12).

As in many other regions around the world (13), there has been a high exposure to psychological distress as well as loneliness and boredom in Tyrol, Austria (approximately 760,000 inhabitants). In our baseline study (conducted between the first and the second wave of the pandemic), 14.4% of 961 participants from the general population scored a GSI-T ≥63 on the Brief-Symptom-Checklist and were classified as psychologically distressed. 22.6% reached a score ≥7 in the Three Item Loneliness Scale and were therefore classified as severely lonely, and boredom levels lay by a mean of 25.9 ± 11.0 points in the Multidimensional State Boredom Scale-Short Form (MSBS-SF) (range: 7–56). Women, single people, and low-income or unemployed people were particularly burdened by these outcomes. Moreover, study participants aged 19–49 reported to be particularly affected by loneliness and boredom (14). Importantly, the relationship between both study participants' sex and partnership situation and psychological distress and severe loneliness was mediated by resilience, and the relationship between participants' partnership situation and psychological distress was mediated by extraversion (15). In addition to investigating longitudinal changes in psychological distress, loneliness, boredom, and resilience over the course of the pandemic, the current 5-month follow-up study aimed at examining the associations between baseline resilience and extraversion on the one hand and psychological distress, loneliness, and boredom at follow-up on the other.

## Methods

The baseline assessment took place from June 26th to August 20th, 2020 by conducting an online survey among adult residents of Tyrol who were recruited through dissemination of a link in different print and social media. Participants were asked to provide an email address in order to be contacted for follow-up, however, providing an email address was not obligatory for participation. The follow-up survey was conducted from November 30th, 2020 to January 24th, 2021. Electronic data collection was performed using the computer-based health evaluation system (CHES) (16). The Ethics Committee of the Medical University of Innsbruck granted ethical approval (no. 1303/2021) and participants provided informed consent online before study participation.

The anonymized survey included sociodemographic and COVID-19-related questions as well as standardized questionnaires. Psychological distress was investigated using the 53-item Brief-Symptom-Checklist (BSCL) which measures nine symptom patterns of mental health problems (17). Items are rated on a 0 (no at all) to 4 (extremely) Likert-scale, the total score ranges from 0 to 212. As recommended by the authors of the BSCL, clinically relevant psychological distress was defined as a Global Severity Index (GSI) T-score ≥63. Loneliness was assessed using the short form of the Revised University of California Los Angeles (R-UCLA) Loneliness Scale, the Three-Item Loneliness Scale (TILS). Scores of 3–9 can be achieved with higher scores indicating more loneliness (18). Scores of 5 or 6 indicate moderate loneliness, and scores ≥7 indicate severe loneliness (19, 20). Boredom was measured with the MSBS-SF. The scale consists of eight Likert-type items that are scored on a seven-point scale. The maximum possible score is 56 with higher scores indicating more boredom (21). Resilience was measured using the Resilience Scale (RS-13), which comprises 13 items on a seven-point Likert scale (1 “strongly disagree”−7 “strongly agree”). The score ranges from 13 to 91 with higher scores indicating higher resilience. A score ≤ 66 reflects low resilience, scores between 67 and 72 indicate moderate resilience, and scores ≥73 indicate high resilience (22). The Extraversion subscale of the Big Five Inventory was used to measure extraversion. This five-point Likert scale (1 “strongly disagree”−5 “strongly agree”) comprises eight items with scores ranging from 8 to 40. The higher the score the more extroverted is the person (23).

### Statistical Methods

Statistical analysis was performed using SPSS, version 26. Changes in sociodemographic variables, COVID-19-related aspects, and psychological scales between baseline and follow-up were analyzed by paired *t*-test, Wilcoxon matched-pairs signed-rank test, or Mc Nemar test, depending on the variable type (normally distributed, not normally distributed, or categorical variables, respectively). Associations of changes in psychological distress, loneliness, and boredom with age, sex, or partnership situation were investigated by non-parametric correlation analysis (Spearman rank correlation), owing to the non-normal distribution of the variables involved. Non-parametric correlation was also applied to analyze associations between baseline scores of resilience and extraversion and follow-up scores of psychological distress, loneliness, and boredom.

### Power Considerations

Under standard assumptions regarding type-one error (alpha = 0.05) and power (1-beta = 0.8), the sample size of 384 is sufficiently large to detect an effect size of *d* = 0.144 by paired *t*-test (*d* = 0.147 by Wilcoxon matched-pairs test). It also allows the detection of odds ratios ≤ 2.5 by a Mc Nemar test, if the proportion of discordant pairs is not too small (≥0.12). The above sample size is also large enough to detect correlation coefficients of *r* = 0.143 or higher. All of these effects are “small” according to Cohen's classification (24).

Moreover, a sample size of 364 persons provides estimates of proportions *p*_0_ with a margin of error of 0.05 or 5%, i.e., the corresponding 95% confidence interval has the form [*p*_0_ – *d, p*_0_ + *d*] with *d* ≤ 0.05. This is an acceptable margin of error for most applications.

## Results

Findings of the baseline survey are presented in Tutzer et al. (14) and Chernova et al. (15). Of the 961 baseline participants, 384 took part in the follow-up survey. Participants who completed both surveys were on average 44.1 ± 14.1 years old. 260 (67.7%) were female, 288 (75%) were in a relationship. The majority (69.8%) of respondents were in permanent employment (full-time or part-time). Just as at baseline (89.9%), the majority (82.7%) of respondents considered the public health policy measures taken against COVID-19 to be useful at follow-up. At baseline, 98.7% adhered to the recommended measures, at follow-up 96.7%.

As shown in Table 1, a significant increase in the consumption of alcohol or other substances in order to feel better was observed over time. The percentage of study participants with striking psychological distress remained the same (15.6 vs. 14.8%). Similarly, resilience did not change from baseline to follow-up, whereas the number of those experiencing moderate loneliness increased significantly from 27.9 to 40.8%. In contrast, at follow-up, severe loneliness was detected in significantly less people (25.5 vs. 20.3%). Boredom decreased significantly over time. Extraversion was only assessed at baseline and participants reached a mean score of 27.9 ± 5.7 points.

**Table 1:** Sociodemographics, COVID-19-related variables, and psychological variables among 384 adults from the general population in the course of time.

**Variable**	**Baseline**	**Follow-up**	**Statistics^a^**	***p*-value**
Work situation				
Full-time or part-time	71.8% (275)	69.8% (265)	χ^2^ = 0.71	0.401
Self-employed	3.1% (12)	2.1% (8)	–^b^	0.388
Education/training	5.7% (22)	3.9% (15)↓	–^b^	0.039
Unemployed	0.8% (3)	2.9% (11)	–^b^	0.057
Retired	12.0% (46)	10.5% (40)	–^b^	0.070
Others	6.5% (25)	10.8% (41)↑	–^b^	0.010
Consumption of alcohol or other substances since the outbreak of the COVID-19 pandemic in order to feel better	19.1% (74)	33.1% (121)↑	χ^2^ = 31.5	<0.001
Did you feel exposed to violence?	1.3% (5)	1.3% (5)	χ^2^ = 0.00	1.000
Has your propensity for violence increased?	8.1% (31)	10.3% (39)	χ^2^ = 1.17	0.379
Psychological distress (*n* = 359)				
BSCL total score	28.0 ± 30.8	28.7 ± 31.2	*Z* = 0.86	0.389
GSI T-score ≥63	15.6% (56)	14.8% (53)	χ^2^ = 0.103	0.749
Loneliness (*n* = 365)				
TILS total score	5.18 ± 1.87	5.19 ± 1.67	*Z* = 0.25	0.803
Moderate (score 5–6)	27.9% (102)	40.8% ↑(149)	χ^2^ = 15.22	<0.001
Severe (score ≥7)	25.5% (93)	20.3% ↓(74)	χ^2^ = 4.10	0.043
Boredom (*n* = 361)				
MSBS-SF total score	25.8 ± 11.1	24.5 ± 11.1↓	*t* = −2.72	0.007
Resilience (RS-13 total, *n* = 367)	71.6 ± 11.6	71.3 ± 12.1	*t* = −0.59	0.555
Extraversion (Big Five subscale, *n* = 381)	27.9 ± 5.7	–^c^	–^c^	–^c^

*BSCL, Brief-Symptom-Checklist; GSI, Global Severity Index; TILS, Three-Item Loneliness Scale; MSBS-SF, Multidimensional State Boredom Scale-Short Form; RS-13, 13-Item Resilience Scale*.

a *McNemar test (χ^2^), paired t-test (t), or Wilcoxon matched pairs signed-rank test (Z) was used, depending on the variable type, see section Statistical Methods*.

b *Exact p-value, based on binomial distribution*.

c *Extraversion was only assessed at baseline*.

*↑Significantly higher than at baseline, p < 0.05*.

*↓Significantly lower than at baseline, p < 0.05*.

Changes in psychological distress (BSCL) or boredom (MSBS-SF) were not significantly correlated with age, sex, or partnership situation. There was a small but statistically significant positive correlation between change in loneliness and age (*r*_*S*_ = 0.151, *p* = 0.004), however, in comparison to the moderate negative correlation of loneliness and age seen at baseline (*r*_*S*_ = −0.275, *p* < 0.001) this effect was small. Similarly, changes in loneliness to the worse were observed in men (mean increase 0.39, *Z* = 2.94, *p* = 0.003, Wilcoxon matched-pairs test), but not in women (mean change −0.12, *p* > 0.1). Again, this effect was small in comparison to the initial difference in TILS total scores between male and female participants (mean TILS scores of 4.76 and 5.34 at baseline in men and women, respectively, difference of 0.58, *p* = 0.009).

Correlations between baseline scores in resilience and extraversion and follow-up scores in psychological distress, loneliness, and boredom are displayed in Table 2. Baseline resilience scores showed a fairly strong inverse relationship with psychological distress, loneliness, and boredom at follow-up (*r* between −0.4 and −0.55). Extraversion exhibited a moderate negative correlation with psychological distress and loneliness and a small but significant correlation with boredom.

**Table 2:** Association of resilience and extraversion at baseline with psychological outcome measures at follow-up (Spearman rank correlation coefficients).

**Measures at baseline**		**Measures at follow-up**		
		**Psychological distress (BSCL total)**	**Loneliness (TILS total)**	**Boredom (MSBS-SF total)**
Resilience (RS-13 total)	*r_*S*_*	−0.546^**^	−0.417^**^	−0.417^**^
	*p*-value (two-tailed)	<0.001	<0.001	<0.001
	*N*	360	365	361
Extraversion (Big Five subscale)	*r_*S*_*	−0.282^**^	−0.214^**^	−0.120^*^
	*p*-value (two-tailed)	<0.001	<0.001	0.022
	*N*	360	365	362

*RS-13, 13-Item Resilience Scale; BSCL, Brief-Symptom-Checklist; TILS, Three-Item Loneliness Scale; MSBS-SF, Multidimensional State Boredom Scale-Short Form; r_S_, Spearman Rank Correlation Coefficient*.

* *p < 0.05*.

** *p < 0.01*.

## Discussion

Reported prevalence of psychological distress among 384 Tyrolean adults was 15.6% at baseline and 14.8% at follow up. Accordingly, psychological burden remained constant despite intermediate reduction of quarantine conditions. Of note, this result corroborates findings from the United States and has been suggested to increase the risk of psychiatric disorders (25). Loneliness also remained unchanged, while boredom decreased significantly. We hypothesize that the decrease in boredom may be a result of the wide range of sports and free time activity programs as well as leisure offers in Tyrol, while loneliness may have remained unchanged due to the continued advice to work from home and various confinements, e.g., travel restrictions, school and university closure, and restaurant closure. Notably, the young population was particularly affected by loneliness. These are highly alarming results since increased loneliness has previously been shown to be associated with higher levels of mental health symptomatology during COVID-19, which, in turn, has been associated with substance use symptoms (26). There was also a significant increase in substance use in our study population. Again, we suggest that the loss of daily structure and family and social support may be of relevance in this regard (27), however, this issue cannot be addressed by our data.

Higher resilience at baseline resulted in significantly lower levels of psychological distress, loneliness, and boredom at follow-up. A recent study from the United States revealed that resilience was significantly lower during the early stage of the COVID-19 pandemic compared to normative data (28). In contrast, no difference in resilience levels was found between baseline and follow-up among our sample, i.e., RS-13 mean scores indicated a constant moderate to high degree of resilience. In the absence of pre-pandemic data it is not possible to determine whether resilience has declined since the onset of the pandemic, however, our results suggest that resilience was unaffected by the subsequent course of the pandemic in the short-term. It remains to be seen whether this changes in the long-term. Further longitudinal data are currently being collected.

Similar to the moderate negative association between baseline resilience and psychological distress, loneliness, and boredom at follow-up we found a weak but still significant negative association between extraversion and these outcomes, which is in line with the findings of Nikčević et al. (29) who described a previously unobserved protective effect of personality traits such as extraversion in predicting psychological stress during the pandemic. They assume that, among others, extraversion contributes to the activation of coping mechanisms and thus mitigates psychological stress (29). A Brazilian study examined extroverts' adherence to social isolation and found that it is a difficult task for extroverts who seek social proximity. Accordingly, extroverts are significantly less likely to adhere to social distancing than others (10), which might reduce their levels of psychological distress during the current pandemic. However, this needs to be investigated in more detail in future studies.

Considering the proven positive effect of resilience and extraversion it should not be forgotten that these two characteristics could be strengthened through targeted training. Resilience training based on mindfulness and/or cognitive and behavioral skills has been associated with improved physical and mental health (30). In addition, research shows that people appear to be able to change certain personality traits at will. For example, extraversion can be strengthened by choosing to reinforce extroverted behavior. If this change in behavior becomes a habit, it can subsequently lead to a change in personality (31). It remains to be seen how such training programs can be implemented in the general population and how such training will affect the long-term well-being of people affected by the COVID-19 pandemic.

This study has some limitations. Recruitment of the current convenience sample was done through advertising and accordingly, individuals who were not reached by this advertising or did not have access to the Internet could not participate. Another limitation affecting the representation of the sample is the uneven distribution in terms of age and gender. As data collection was anonymous, it's not possible to determine whether the participants originate from a specific sub-region. Accordingly, one has to be cautious in generalizing the results, as sample bias may have occurred. Similarly, due to the study design, all data are based on self-reporting by participants, which may lead to social desirability bias. In addition, we did not collect data on disabilities which may be associated with increased loneliness in the context of the pandemic (32). This issue therefore cannot be addressed by our data. Despite these limitations and although we did not collect pre-pandemic data, the longitudinal design of the study is a strength. Further follow-up surveys will allow the investigated issues to be explored over the entire duration of the pandemic.

In conclusion, these results display a high psychological burden over the course of the pandemic in a subgroup of the general population. Obviously, a slight relaxation of confinements is not sufficient to reduce psychological distress, loneliness, and boredom. Since the further course of the pandemic and its end are uncertain, our findings reemphasize that it is all the more important to take preventive measures aiming at strengthening resilience and extraversion.

## Data Availability Statement

The original contributions presented in the study are included in the article/supplementary files, further inquiries can be directed to the corresponding author/s.

## Ethics Statement

The studies involving human participants were reviewed and approved by the Ethics Committee of the Medical University Innsbruck. The patients/participants provided their online informed consent to participate in this study.

## Author Contributions

AH, BF-A, SP, BH, and BP designed the study and wrote the protocol. Recruitment was performed by FT and AC. GK undertook statistical analysis and FT wrote the first draft of the manuscript. All authors contributed to and have approved the final manuscript.

## Funding

This work was supported by a grant (no. F.21427) from the Federal State of Tyrol.

## Conflict of Interest

BH owns part of the IPRs of the CHES software tool. The remaining authors declare that the research was conducted in the absence of any commercial or financial relationships that could be construed as a potential conflict of interest.

## Publisher's Note

All claims expressed in this article are solely those of the authors and do not necessarily represent those of their affiliated organizations, or those of the publisher, the editors and the reviewers. Any product that may be evaluated in this article, or claim that may be made by its manufacturer, is not guaranteed or endorsed by the publisher.

## References

[1] Hossain MM Tasnim S Sultana A Faizah F Mazumder H Zou L. Epidemiology of mental health problems in COVID-19: a review. F1000Res. (2020). 9:636. 10.12688/f1000research.24457.1 33093946 PMC7549174

[2] Brooks SK Webster RK Smith LE Woodland L Wessely S Greenberg N. The psychological impact of quarantine and how to reduce it: rapid review of the evidence. Lancet. (2020) 395:912–20. 10.1016/S0140-6736(20)30460-8 32112714 PMC7158942

[3] Yan L Gan Y Ding X Wu J Duan H. The relationship between perceived stress and emotional distress during the COVID-19 outbreak: effects of boredom proneness and coping style. J Anxiety Disord. (2020) 77:102328. 10.1016/j.janxdis.2020.102328 33160275 PMC7598556

[4] Goldberg Y Eastwood J LaGuardia J Danckert J. Boredom: an emotional experience distinct from apathy, anhedonia, or depression. J Soc Clin Psychol. (2011) 30:647–66. 10.1521/jscp.2011.30.6.647

[5] Wilson RS Krueger KR Arnold SE Schneider JA Kelly JF Barnes LL. Loneliness and risk of Alzheimer disease. Arch Gen Psychiatry. (2007) 64:234–40. 10.1001/archpsyc.64.2.234 17283291

[6] Stravynski A Boyer R. Loneliness in relation to suicide ideation and parasuicide: a population-wide study. Suicide Life Threat Behav. (2001) 31:32–40. 10.1521/suli.31.1.32.21312 11326767

[7] Lutha SS Cicchetti D. The construct of resilience: implications for interventions and social policies. Dev Psychopathol. (2000) 12:857–85. 10.1017/S0954579400004156 11202047 PMC1903337

[8] Haddadi P Besharat MA. Resilience, vulnerability and mental health. Proc Soc Behav Sci. (2010) 5:639–42. 10.1016/j.sbspro.2010.07.157

[9] McCabe KO Fleeson W. What is extraversion for? Integrating trait and motivational perspectives and identifying the purpose of extraversion. Psychol Sci. (2012) 23:1498–505. 10.1177/0956797612444904 23104678 PMC3891893

[10] Carvalho LF Pianowski G Gonçalves AP. Personality differences and COVID-19: are extroversion and conscientiousness personality traits associated with engagement with containment measures? Trends Psychiatry Psychother. (2020) 42:179–84. 10.1590/2237-6089-2020-0029 32294713

[11] Gong Y Shi J Ding H Zhang M Kang C Wang K. Personality traits and depressive symptoms: the moderating and mediating effects of resilience in Chinese adolescents. J Affect Disord. (2020) 265:611–7. 10.1016/j.jad.2019.11.102 31787420

[12] Morales-Vives F Dueñas J-M Vigil-Colet A Camarero-Figuerola M. Psychological variables related to adaptation to the COVID-19 lockdown in Spain. Front Psychol. (2020). 11:565634. 10.3389/fpsyg.2020.565634 33041929 PMC7525044

[13] Talevi D Socci V Carai M Carnaghi G Faleri S Trebbi E. Mental health outcomes of the CoViD-19 pandemic. Riv Psichiatr. (2020) 55:137–44. 10.1708/3382.33569 32489190

[14] Tutzer F Frajo-Apor B Pardeller S Plattner B Chernova A Haring C. Psychological distress, loneliness, and boredom among the general population of Tyrol, Austria during the COVID-19 pandemic. Front Psychiatry. (2021). 12:691896. 10.3389/fpsyt.2021.691896 34177672 PMC8222609

[15] Chernova A Frajo-Apor B Pardeller S Tutzer F Plattner B Haring C. The mediating role of resilience and extraversion on psychological distress and loneliness among the general population of Tyrol, Austria between the first and the second wave of the COVID-19 pandemic. Front Psychiatry. (2021) 12:766261. 10.3389/fpsyt.2021.766261 34777068 PMC8578839

[16] Holzner B Giesinger JM Pinggera J Zugal S Schopf F Oberguggenberger AS. The Computer-based Health Evaluation Software (CHES): a software for electronic patient-reported outcome monitoring. BMC Med Inform Decis Mak. (2012) 12:126. 10.1186/1472-6947-12-126 23140270 PMC3529695

[17] Franke GH. BSCL: Brief-Symptom-Checklist. Göttingen: Hogrefe (2017).

[18] Hughes ME Waite LJ Hawkley LC Cacioppo JT. A short scale for measuring loneliness in large surveys: results from two population-based studies. Res Aging. (2004) 26:655–72. 10.1177/0164027504268574 18504506 PMC2394670

[19] Boehlen F Herzog W Quinzler R Haefeli WE Maatouk I Niehoff D. Loneliness in the elderly is associated with the use of psychotropic drugs. Int J Geriatr Psychiatry. (2015) 30:957–64. 10.1002/gps.4246 25504324

[20] Lasgaard M Friis K Shevlin M. “Where are all the lonely people?” A population-based study of high-risk groups across the life span. Soc Psychiatry Psychiatr Epidemiol. (2016) 51:1373–84. 10.1007/s00127-016-1279-3 27571769

[21] Hunter JA Dyer KJ Cribbie RA Eastwood JD. Exploring the utility of the Multidimensional State Boredom Scale. Eur J Psychol Assess. (2016) 32:241–50. 10.1027/1015-5759/a000251

[22] Leppert K Koch B Brähler E Strauss B. Die Resilienzskala (RS) – überprüfung der langfrom RS-25 und einer kurzform RS-13. Klin Diagn Eval. (2008) 1:226–43.

[23] Lang F Lüdtke O Asendorpf J. Testgüte und psychometrische äquivalenz der deutschen version des Big Five Inventory (BFI) bei jungen, mittelalten und alten erwachsenen. Diagnostica. (2001) 47:111–21. 10.1026//0012-1924.47.3.111

[24] Cohen J. Statistical Power Analysis for the Behavioral Sciences. 2nd ed. New York, NY: Routledge (1998).

[25] McGinty EE Presskreischer R Anderson KE Han H Barry CL. Psychological distress and COVID-19-related stressors reported in a longitudinal cohort of US adults in April and July 2020. JAMA. (2020) 324:2555–7. 10.1001/jama.2020.21231 33226420 PMC7684524

[26] Horigian VE Schmidt RD Feaster DJ. Loneliness, mental health, and substance use among US young adults during COVID-19. J Psychoactive Drugs. (2021) 53:1–9. 10.1080/02791072.2020.1836435 33111650

[27] The Lancet Gastroenterology H. Drinking alone: COVID-19, lockdown, and alcohol-related harm. Lancet Gastroenterol Hepatol. (2020) 5:625. 10.1016/S2468-1253(20)30159-X 32553138 PMC7295462

[28] Killgore WDS Taylor EC Cloonan SA Dailey NS. Psychological resilience during the COVID-19 lockdown. Psychiatry Res. (2020) 291:113216. 10.1016/j.psychres.2020.113216 32544705 PMC7280133

[29] Nikčević AV Marino C Kolubinski DC Leach D Spada MM. Modelling the contribution of the Big Five personality traits, health anxiety, and COVID-19 psychological distress to generalised anxiety and depressive symptoms during the COVID-19 pandemic. J Affect Disord. (2021) 279:578–84. 10.1016/j.jad.2020.10.053 33152562 PMC7598311

[30] Joyce S Shand F Tighe J Laurent SJ Bryant RA Harvey SB. Road to resilience: a systematic review and meta-analysis of resilience training programmes and interventions. BMJ Open. (2018). 8:e017858. 10.1136/bmjopen-2017-017858 29903782 PMC6009510

[31] Margolis S Lyubomirsky S. Experimental manipulation of extraverted and introverted behavior and its effects on well-being. J Exp Psychol Gen. (2020) 149:719–31. 10.1037/xge0000668 31368759

[32] Heinze N Hussain SF Castle CL Godier-McBard LR Kempapidis T Gomes RSM. The long-term impact of the COVID-19 pandemic on loneliness in people living with disability and visual impairment. Front Public Health. (2021). 9:738304. 10.3389/fpubh.2021.738304 34568266 PMC8458570

